# Antibodies to Lactobacilli and Bifidobacteria in Young Children with Different Propensity to Develop Islet Autoimmunity

**DOI:** 10.1155/2014/325938

**Published:** 2014-03-04

**Authors:** Ija Talja, Anna-Liisa Kubo, Riitta Veijola, Mikael Knip, Olli Simell, Jorma Ilonen, Mari Vähä-Mäkilä, Epp Sepp, Marika Mikelsaar, Meeme Utt, Raivo Uibo

**Affiliations:** ^1^Immunology Group, Institute of Biomedicine and Translational Medicine, University of Tartu, Ravila 19, 50411 Tartu, Estonia; ^2^Department of Pediatrics, University of Oulu, Kajaanintie 50, 90014 Oulu, Finland; ^3^Children's Hospital, University of Helsinki and Helsinki University Central Hospital, Tukholmankatu 8 A, 00029 Helsinki, Finland; ^4^Department of Pediatrics, Tampere University Hospital, Teiskontie 35, 33521 Tampere, Finland; ^5^Folkhälsan Research Center, Haartmansg 8, 00290 Helsinki, Finland; ^6^Department of Pediatrics, University of Turku, 20014 Turku, Finland; ^7^Immunogenetics Laboratory, University of Turku, 20014 Turku, Finland; ^8^University of Eastern Finland, Yliopistonranta 1, 70211 Kuopio, Finland; ^9^Department of Microbiology, University of Tartu, Ravila 19, 50411 Tartu, Estonia; ^10^Centre for Translational Medicine, University of Tartu, Ravila 19, 50411 Tartu, Estonia

## Abstract

The intestinal microbiota is essential to the maturation and homeostasis of the immune system. Immunoblot assays were used to establish the prevalence of serum IgG, IgM, and IgA antibodies specific for *Bifidobacterium adolescentis*, *Bifidobacterium longum*, and *Lactobacillus rhamnosus* GG proteins in young children presenting with or without type 1 diabetes (T1D). We demonstrated that children between the ages of 6 and 12 months had a substantial increase in the frequency of IgG antibodies specific for *L. rhamnosus* GG proteins. We measured IgG, IgM, and IgA class antibody reactivity against *B. adolescentis* DSM 20083, *B. adolescentis* DSM 20086, and *B. longum* DSM 20088 proteins demonstrating significantly higher IgA responses against *B. adolescentis* DSM 20083 strain proteins in children who developed islet autoimmunity and T1D later in life. *B. adolescentis* strains showed more IgM type antibodies in children who developed T1D later in life, but the difference was not statistically significant. *B. longum* proteins were recognized by IgG and IgA antibodies to a higher extent compared to other bacteria studied. These results confirm that differences in immune reactivity against some commensal strains in young children may represent a different risk factor for developing T1D.

## 1. Introduction

Type 1 diabetes (T1D) is characterized by immune-mediated destruction of the insulin-secreting *β* cells in the pancreatic islets as a result of an unknown trigger mechanism. It is, however, well known that development of clinical disease is preceded by an asymptomatic latent period during which immune reactions against the insulin-secreting cell autoantigens can be demonstrated [1–3]. In this context, biochemically detectable autoantibodies against insulin (IAA), glutamic acid decarboxylase (GADA), insulinoma-associated antigen 2 (IA-2A), and Zn-transporter 8 (ZnT8A) as well as their counterpart immunofluorescent anti-islet antibodies (ICA) serve as reliable biomarkers for T1D development. Specifically, Knip et al. [3] demonstrated that all children initially testing positive for both GADA and IA-2A progressed to clinical T1D over a 26-year followup.

Over the last few decades the incidence of T1D has dramatically increased in many countries particularly in early childhood, suggesting that an event associated with progression towards T1D disease was occurring early in life. An increasing number of studies have suggested that the composition of the intestinal microbiota might contribute significantly to the development of disorders such as T1D since changes to the microflora mirror changes in general life styles and the social system [4–6].

It is believed that intestinal colonization with certain bacteria strongly influences systemic immune responses early in life and may play a significant role in modulating the development of various chronic diseases [7]. Some of the most common constituents of the gastrointestinal tract microbiota include *Bifidobacterium* and *Lactobacillus* species that have been shown to play a significant role in the development of immune-mediated disorders in humans [8–11]. That is, predominant colonization with *B. adolescentis* has been reported in patients with allergic disorders compared to colonization patterns observed in individuals with nonallergic disorders [12–14]. Other *Bifidobacterium* species have been shown to have diverse effects, including variable associations of *B. longum* with immune-mediated and inflammatory diseases. Studies of rodent disease models [15, 16] have also identified differences in the ability of different *Bifidobacterium* species in modulating immune reactivity and inflammation. These observations are in line with study results showing that different *Lactobacillus* spp. may have diverse immunomodulating effects on different diseases [17]. Most well known are the effects of the probiotic strain *L. rhamnosus* GG in preventing atopic eczema among infants, possibly by modulating the immune response to allergens [18]. The recent identification of the *L. rhamnosus* GG p40 molecule as an immunomodulator [19] represents a significant step forward towards resolving problems related to the effects of probiotics *in vivo*.

Previously we demonstrated that the elicitation of antibodies against *L. acidophilus* antigenic components differed between children with various chronic diseases [20]. The current study describes experiments designed to extend these observations by investigating the prevalence of serum antibodies against *B. adolescentis*, *B. longum*, and *L. rhamnosus* GG in young children that developed or did not develop T1D.

## 2. Material and Methods

### 2.1. Plasma Samples

Plasma samples (*n* = 107) from 38 children participating in the Finnish Type 1 Diabetes Prediction and Prevention (DIPP) study and born between 1995 and 2003 were included in this study. Children were separated into 2 groups of 19 children (11 females) each matched for age and sex. One group was comprised of children who later developed at least 2 T1D-related autoantibodies and subsequently clinical T1D (islet autoimmunity [IA], i.e., the IA-positive group) and the other group was comprised of children that did not develop or present with signs of islet autoimmunity (IA-negative group) and without T1D during followup. Islet autoimmunity was defined in this context as detection of at least 2 antibodies out of GADA (assay sensitivity 82%, specificity 96%), IA-2A (assay sensitivity 72%, specificity 100%), and/or ICA. Levels of ICA were measured by an indirect immunofluorescence assay with a detection limit of 2.5 Juvenile Diabetes Foundation Units. All children in the IA-positive group later developed T1D (age at onset ranged between 2.4 and 10.3 years). Both groups were similar in their documented use of antibiotics (during the first 2 years of life 13/18 IA-positive children and 17/18 IA-negative children were treated with antibiotics; *P* = 0.177; the data pertaining to one child from each group was not available). No differences in the use of probiotics between groups were observed (2 children from the IA-positive group; *P* = 0.487).

### 2.2. Bacterial Strains and Cell Lysate Preparation

Wilkins-Chalgren agar (Oxoid, UK) was used to culture *B. adolescentis* DSM 20083 (ATCC 15703) and DSM 20086 (ATCC 15705) and *B. longum* DSM 20088 (ATCC 15697). Man-Rogosa-Sharpe agar (Oxoid, UK) was used to culture *L. rhamnosus* GG. Wilkins-Chalgren agar plates were incubated in an anaerobic cabinet (Concept, UK; with gas mixture of 5% CO_2_, 5% H_2_, and 90% N_2_) and Man-Rogosa-Sharpe agar in a microaerobic environment (Joan, France) with a gas mixture of 10% CO_2_ for 48 h.

Bacterial cells were collected, suspended in phosphate-buffered saline solution (PBS, pH = 7.4), and washed 3 times with the same buffer. Subsequently, cells were disrupted with 0.1 mm glass beads (Biospec Products, USA) in PBS in the presence of complete protease inhibitors (Boehringen Mannheim-Roche, Switzerland) on ice. The total protein concentration in lysates was determined using the Protein Assay solution (Bio-Rad, USA) using bovine serum albumin as a standard and kept in aliquots at −20°C until used.

### 2.3. Gradient Sodium Dodecyl Sulphate Polyacrylamide Gel Electrophoresis (SDS-PAGE) and 1D Immunoblotting

Equal amounts of proteins from different *Bifidobacteria* spp. preparations were mixed with 300 *μ*L SDS-PAGE sample buffer and heated for 15 min at 95°C. To each gel approximately 100 *μ*g of protein was loaded.

The proteins in the bacterial cell lysate were loaded on a 5–20% gradient gel with a 5% concentrating gel with All Blue 10–250 kDa molecular weight (MW) markers (Bio-Rad, USA) as standards and separated by electrophoresis using a current of 40 mA and a voltage of 200 V for 6 h using a vertical electrophoresis system SE-600 (Hoefer, USA) connected to a thermostatic circulator. Separated proteins were transferred onto a polyvinylidene difluoride (PVDF) membrane (0.45 *μ*m pore size) using a semidry electroblotter (Hoefer, USA) at a current density of 1 mA/cm^2^ for 1.5 h and membranes blocked as described by Nilsson et al. [21].

Five mm wide strips were cut from the membrane and incubated with plasma samples diluted to 1 : 50 for IgA and IgM and 1 : 100 for IgG in the incubating buffer [21] overnight at 4°C. Strips were then incubated with either secondary anti-human IgA, anti-human IgM, or anti-human IgG antibodies labelled with horse-radish peroxidase (HRP) (diluted 1 : 500; Dako, Denmark) for 1 h and subsequently developed in substrate solution comprised of 0.04% carbazole in 50 mM sodium acetate buffer (pH = 5.0) and hydrogen peroxide (0.015%) for 30 min at room temperature. All serum samples were screened in similar fashion. The strips were scanned using a Bio-Rad GS-710 Imaging densitometer (Bio-Rad, USA). The relative MW of the bands was estimated with the Bio-Rad Quantity One image analysis software (Bio-Rad, USA) according to the All Blue molecular weight markers.

### 2.4. Statistical Analysis

The *F*-test was used to compare the frequency of antibodies in the two groups. *P* values below 0.05 were considered statistically significant.

## 3. Results

### 3.1. IgG Antibody Reactivity to *L. rhamnosus* GG Proteins

We first analyzed the dynamics of IgG antibodies that developed against *L. rhamnosus* GG target antigens in children between 3 and 24 months of age. This approach was chosen due to the frequent consumption of the probiotic *L. rhamnosus* GG-containing dairy products by children and adults in Finland. We discovered 25 clearly distinguishable *L. rhamnosus* GG antigenic proteins ranging between 11 and 86 kDa (Table 1). The number of* L. rhamnosus* GG proteins recognized by IgG increased steadily with age.  Table 2 describes the 9 most frequently reactive proteins. Children between the ages of 12 and 24 months presented with significantly more antibodies against 5/9 (31, 42, 45, 47, and 49 kDa proteins) selected highly reactive proteins compared to reactivity observed in 3-month-old infants. However, no significant differences in IgG reactivity were observed in response to *L. rhamnosus* GG antigens between IA-positive and IA-negative children.

The average number of IgG-reactive *L. rhamnosus* GG protein bands was 10.5 ± 6.6 in IA-positive infants (between 5 and 17 bands) and 11.5 ± 7.2 in IA-negative children (between 5 and 20 bands) at 12 months of age. Based on these results the 12-month-old group was chosen for further analysis of immune reactivity against *Bifidobacterium* spp. antigens.

### 3.2. IgG Reactivity against *B. adolescentis* and *B. longum* Proteins

**Figure 1:**
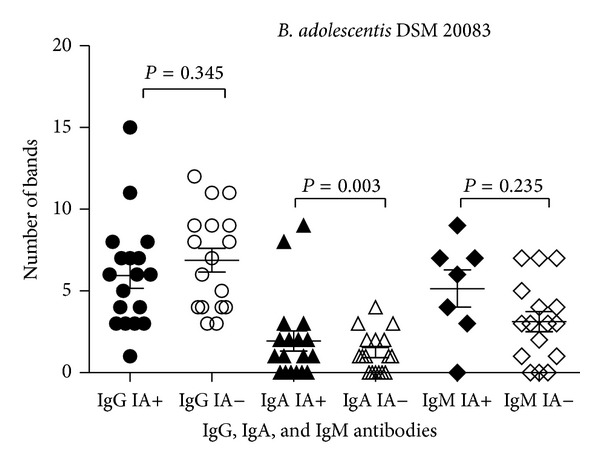
IgG, IgA, and IgM reactivity to *B. adolescentis* DSM 20083 proteins among IA-positive and IA-negative individuals.

**Figure 2:**
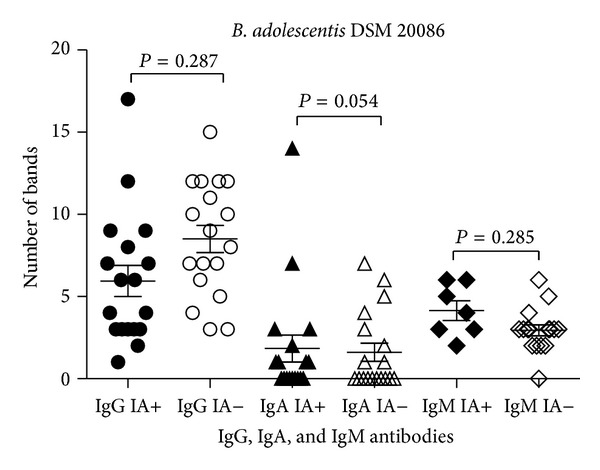
IgG, IgA, and IgM reactivity to *B. adolescentis* DSM 20086 proteins among IA-positive and IA-negative individuals.

**Figure 3:**
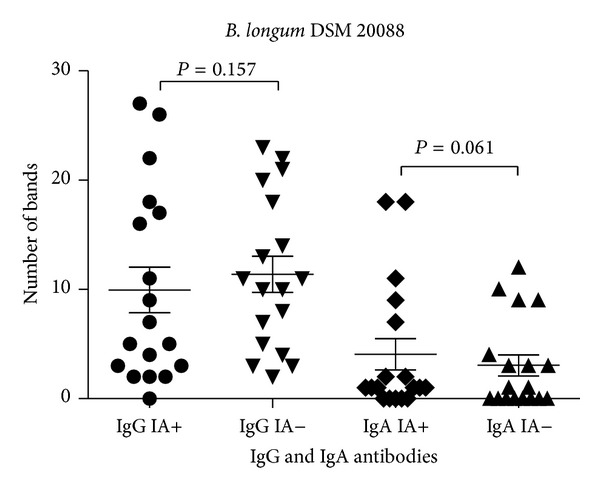
IgA and IgG reactivity against *B. longum* DSM 20088 proteins among IA-positive and IA-negative individuals.

IgG bound to *Bifidobacterium* antigens in the molecular weight ranging from 7 kDa to 131 kDa. The number of proteins recognized varied between strains with the greatest number of reactive antigens observed for *B. longum* DSM 20088 (*N* = 57; Table 1). The average number of IgG-reactive proteins in assays with *B. adolescentis* DSM 20083, *B. adolescentis* DSM 20086, and *B. longum* DSM 20088 strains was not significantly different between IA-positive children and IA-negative subjects (Figures 1–3).

### 3.3. IgA Reactivity against *B. adolescentis* and *B. longum* Proteins

IgA bound to protein antigens in the molecular weight ranging from 7 kDa to 104 kDa (Table 1). Similar to the IgG antibody results, more *B. longum* DSM 20088 proteins were recognized by IgA than any other tested bacteria. The average number of *B. adolescentis* DSM 20083 proteins bound by IgA was lower than the number of proteins bound from other strains. There was a significant difference in the number of proteins reactive to antibodies between the 2 groups of children: 1.9 ± 2.6 reactive protein bands in IA-positive infants and 1.2 ± 1.2 reactive protein bands in IA-negative children (*P* < 0.003; Figure 1). Also in tests with *Bifidobacterium* strains the average number of reacting antigenic proteins tended to be at a higher frequency in the IA-positive group compared to the IA-negative group (Figures 2 and 3).

### 3.4. IgM Reactivity to *B. adolescentis* Proteins

IgM bound to bacterial proteins in the molecular weight ranging from 13 kDa to 84 kDa. The average number of IgM reacting protein bands in both assays either with *B. adolescentis* DSM 20083 or with *B. adolescentis* DSM 20086 was higher in IA-positive than in IA-negative infants, but this difference was not statistically significant (*P* = 0.235 and *P* = 0.285, resp.; Figures 1 and 2).

## 4. Discussion

This study investigated the reactivity of serum antibodies against the probiotic strain *L. rhamnosus* GG and selected natural *Bifidobacterium* spp. proteins in children 3, 6, 12, and 24 months of age using an immunoblot assay developed previously by our group [20]. *L. rhamnosus* GG is included into the most commonly used dairy products in Finland, whereas all chosen Bifidobacterium strains, *B. adolescentis* DSM 20083, *B. adolescentis* DSM 20086, and *B. longum* DSM 20088, are usual inhabitants of the intestinal microbiota. The colonization of newborns with microbes begins during delivery and by 2 months of age 91% of infants are colonized with *B. longum*, 75% with *B. adolescentis*, and 57% with Lactobacilli group I (*L. rhamnosus*, *L. casei*, and *L. paracasei*) [10]. A shift in colonization with *B. adolescentis* strains towards earlier life has been demonstrated recently [14] and this has also been observed at the age of 5 years [13].

The population studied here was obtained from the Finnish DIPP Study and divided into 2 groups based on the development of diabetes-associated autoantibodies and T1D over a 10-year followup. Immunoblot analyses of bacterial lysates demonstrated a substantial increase in the frequency of IgG antibody reactivity to *L. rhamnosus* GG proteins between 6 and 12 months of age, showing that the most conspicuous increase in antibody production occurred before the age of 12 months. In spite of these age-dependent changes, reactions against *L. rhamnosus* GG proteins were seen in IA-positive and IA-negative subjects at a similar frequency. This result indicated that IgG antibodies reactive to probiotic strain *L. rhamnosus* GG proteins were not suggestive of progression toward *β*-cell autoimmunity or development of T1D in children. The most important observation made from immunoblot assays testing antibody reactivity to *L. rhamnosus* GG proteins was the age-related dynamics associated with the development of circulating antibodies to microbial proteins. This emphasized how important it was to carry out these analyses using *Bifidobacterium* spp. proteins since these organisms are known to colonize the infant's gut early in life. We assessed binding to different antigenic proteins from *B. adolescentis* DSM 20083, *B. adolescentis* DSM 20086, and *B. longum* DSM 20088 by IgG, IgA, and IgM class antibodies and observed that IgA bound significantly more *B. adolescentis* DSM 20083 strain antigens in children who developed islet autoimmunity and T1D later in life. At the same time we could not find any significant differences between the two groups of children in the frequencies of IgG or IgA antibodies against other bifidobacteria strains. It is noteworthy that we found that *B. longum* proteins were recognized by IgG and IgA antibodies to a substantially higher extent compared to other bacteria studied. This phenomenon may indicate a stronger stimulation of the infant's immune system by *B. longum* protein antigens.

Some of the immunoreactive bands detected may be due to cross-reactive antibodies that developed in response to bacterial antigens from species not used in this study. It is however very difficult to make this distinction since we presently have no tools (monoclonal antibodies) to discriminate between strain-specific reactivity and cross-reactivity. Regardless, it was clear that differences in the development of IgA antibodies with reactivity against *B. adolescentis* DSM 20083 (ATCC 15703) antigens were present independent of the mechanism involved. It is of particular importance to note that differences between the 2 groups were associated with reactivity by IgA but not IgG or IgM class antibodies. IgA with reactivity against commensal intestinal microbes can develop as a consequence of colonization with high numbers of organisms at the mucosal surface [22]. It has been shown that oral administration of the probiotic mixture VSL#3 that includes 8 different probiotic strains (including 3 bifidobacteria strains) prevented the development of autoimmune diabetes in nonobese diabetic mice [23]. Different bifidobacteria strains have clearly been shown to have variable influences on immune responses [23, 24]. The species of *B. adolescentis* most commonly isolated from allergic children has been shown to more effectively trigger the production of proinflammatory cytokines compared to other strains of bifidobacteria [25, 26] supporting our results that demonstrated differences between children with and without signs of *β*-cell autoimmunity with regard to reactivity to *B. adolescentis* strain 20083 antigens which might impact T1D development. Whether these differences are in some way linked to differences in the microbiome needs further studies [27, 28].

In conclusion, during the first year of life the immune system starts to produce antibodies which recognize antigens produced by organisms comprising the normal microbiota. Although some of these proteins may contain common antigenic epitopes present on proteins produced by various bacteria, our current study showed that differences in antibody reactivities could be found even between the same species of *B. adolescentis*. These differences might reflect a propensity for developing T1D confirming recent findings regarding differences in the gut microbiome between children affected and unaffected by T1D. Further studies will be needed to verify how the presence of this particular *B. adolescentis* DSM 20083 strain in the intestine of children is involved in predisposing individuals to developing *β*-cell autoimmunity and T1D.

## Figures and Tables

**Table 1:** Molecular weight ranges of proteins reacting with IgA and IgG antibodies and the number of detectable antigenic proteins present in different bacterial homogenates.

	Antigenic protein MW ranges		Number of reactive antigenic proteins	
	IgG	IgA	IgG	IgA
*L. rhamnosus* GG	11–86 kDa	n.t.*	25	n.t.*
*B. adolescentis* DSM 20083	7–80 kDa	7–72 kDa	31	20
*B. adolescentis* DSM 20086	10–131 kDa	10–92 kDa	36	24
*B. longum* DSM 20088	10–127 kDa	10–104 kDa	57	44

*n.t.: not tested.

**Table 2:** The number of children in different age groups with serum IgG reactivity to different *L. rhamnosus *GG proteins. The age period when significantly more (*P* < 0.05) antibodies against bacterial proteins were observed compared to the 3-month age group is shown in bold.

*L. rhamnosus* GG antigenic proteins	3-month		6-month		12-month		24-month	
	IA-positive	IA-negative	IA-positive	IA-negative	IA-positive	IA-negative	IA-positive	IA-negative
	(*N* = 18)	(*N* = 18)	(*N* = 19)	(*N* = 19)	(*N* = 18)	(*N* = 18)	(*N* = 15)	(*N* = 18)
65 kDa	8 (44)	14 (78)	14 (74)*	**17 (89)**	**17 (94) **	**17 (94) **	**15 (100)**	**17 (94)**
58 kDa	8 (44)	12 (67)	**18 (95) **	**19 (100) **	**17 (94)**	**17 (94)**	**15 (100)**	**18 (100)**
49 kDa	2 (11)	2 (11)	2 (10)	4 (21)	**6 (33)**	**8 (44)**	**4 (27) **	**7 (39)**
47 kDa	4 (22)	4 (22)	8 (42)	7 (37)	**9 (50)**	**12 (67)**	**7 (47)**	**8 (44)**
45 kDa	9 (50)	11 (61)	11 (58)	11 (58)	**16 (89)**	**14 (78)**	**15 (100)**	**18 (100)**
42 kDa	3 (17)	4 (22)	5 (26)	7 (37)	**9 (50)**	**7 (39)**	**8 (53)**	**9 (50)**
38 kDa	1 (5)	3 (17)	2 (10)	2 (10)	5 (28)	5 (28)	**8 (53)**	**8 (44)**
31 kDa	13 (72)	9 (50)	14 (74)	11 (58)	**17 (94)**	**17 (94)**	**15 (100)**	**16 (89) **
27 kDa	0 (0)	4 (22)	1 (5)	2 (10)	2 (11)	2 (11)	**5 (33)**	**6 (33)**

*Number and % (in brackets) of children with positive reactions to this protein.
